# East Antarctica magnetically linked to its ancient neighbours in Gondwana

**DOI:** 10.1038/s41598-021-84834-1

**Published:** 2021-03-09

**Authors:** Jörg Ebbing, Yixiati Dilixiati, Peter Haas, Fausto Ferraccioli, Stephanie Scheiber-Enslin

**Affiliations:** 1 Institute for Geosciences, Kiel University, Kiel, Germany; 2 OGS, Trieste, Italy; 3 British Antarctic Survey, Cambridge, UK; 4 Witwatersrand University, Johannesburg, South Africa

**Keywords:** Geomagnetism, Geophysics, Tectonics

## Abstract

We present a new magnetic compilation for Central Gondwana conformed to a recent satellite magnetic model (LCS-1) with the help of an equivalent layer approach, resulting in consistent levels, corrections that have not previously been applied. Additionally, we use the satellite data to its full spectral content, which helps to include India, where high resolution aeromagnetic data are not publically available. As India is located north of the magnetic equator, we also performed a variable reduction to the pole to the satellite data by applying an equivalent source method. The conformed aeromagnetic and satellite data are superimposed on a recent deformable Gondwana plate reconstruction that links the Kaapvaal Craton in Southern Africa with the Grunehogna Craton in East Antarctica in a tight fit. Aeromagnetic anomalies unveil, however, wider orogenic belts that preserve remnants of accreted Meso- to Neoproterozoic crust in interior East Antarctica, compared to adjacent sectors of Southern Africa and India. Satellite and aeromagnetic anomaly datasets help to portray the extent and architecture of older Precambrian cratons, re-enforcing their linkages in East Antarctica, Australia, India and Africa.

## Introduction

**Figure 1:**
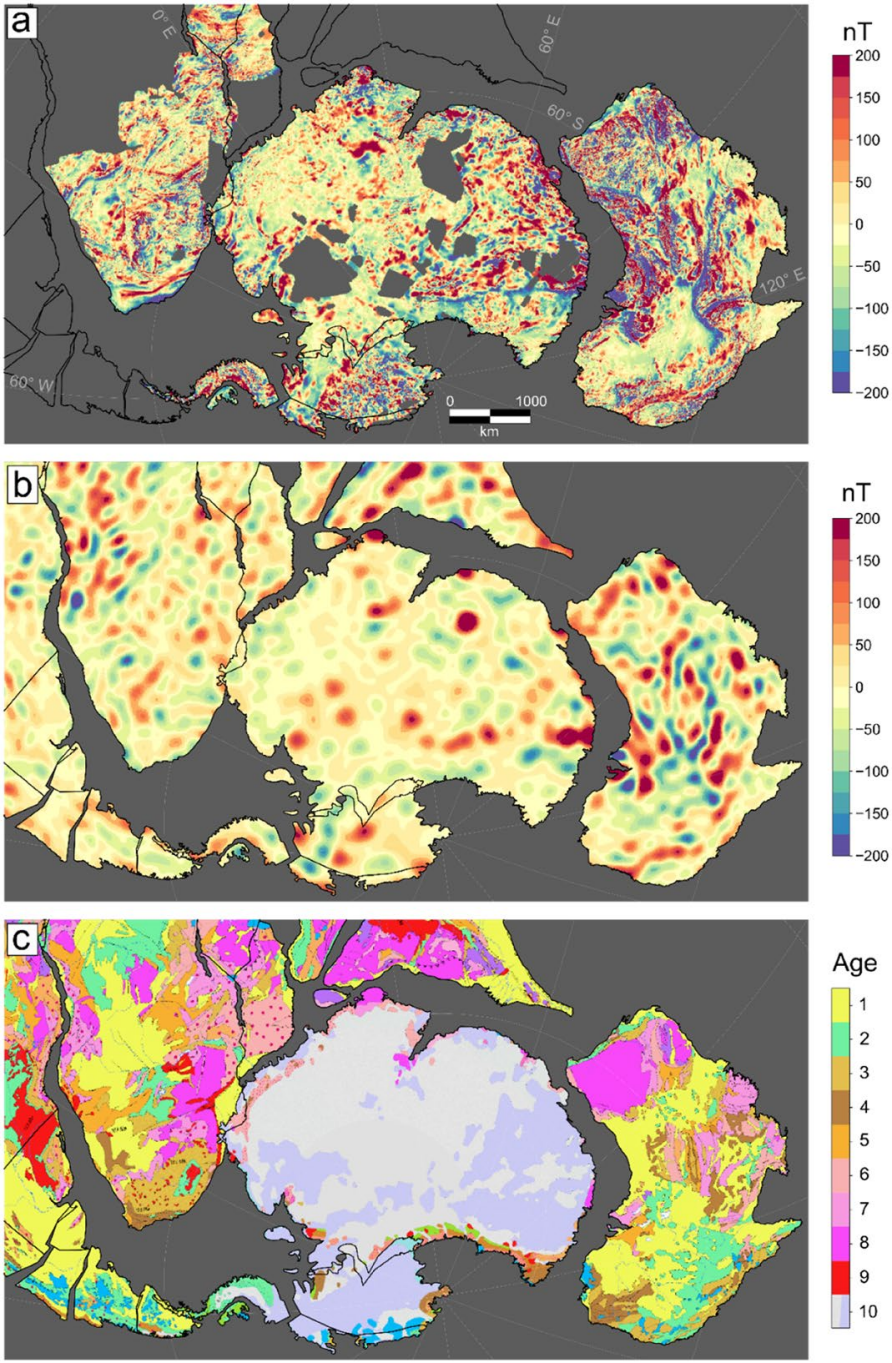
(**A**) Conformed aeromagnetic data, (**B**) Satellite model LCS-1 after variable reduction to the pole, represented at 5 km ellipsoidal height. (**C**) Surface geology as represented in the Geological Map of the World^28^. The geological units are: 1: Cenozoic, 2: Mesozoic, 3: Upper Paleozoic, 4: Lower Paleozoic, 5: Neoproterozoic, 6: Mesoproterozoic, 7: Paleoproterozoic, 8: Archean, 9: Large Igneous Provinces, 10: Glaciers and Ice. For more details, see^28^. All data sets are rotated back to 200 Ma with the deformable plate model^5^.

Defining the architecture of the lithosphere under the thick ice cover of Antarctica is of wide interest as large parts are still blanks on geological maps (Fig. 1 and recent Gondwana map^1^). Novel methods and data are needed to enhance our current knowledge and link the geology from the few outcrops at the coast to that under the ice^2,3^. For example, in a recent study, heat-flow values from the formerly adjacent neighbours were interpolated into East Antarctica, deriving a map significantly different from previous interpretations^4^. A second study uses a set of geophysical data and models to define lithospheric domains in East Antarctica by statistical analysis^3^. Both these statistical methods in their simplicity provide new insights, but have limitations, mostly due to the sparse coverage of data used.

Magnetic data collected over the last 50 years, although with varying resolution and accuracy, covers Antarctica and its ancient continental neighbours. Previous studies have shown that magnetic data, especially when interpreted jointly with geochronological, geochemical, geophysical and palaeomagnetic datasets, helps identifying fundamental links between formerly adjacent neighbours within the Gondwana, Rodinia and Columbia supercontinents^5,6^. These links are highly relevant for global supercontinent studies as current plate reconstructions still vary considerably, both in terms of defining the relative positions and the tectonic processes that affected different continents.

In this context, Antarctica has a special role as it holds a key position in a Gondwana framework^7–9^ but its geology is mostly hidden under its thick ice cover. Therefore, reconstructing the position of Antarctica helps to extrapolate the geology from the former adjacent neighbours. For example, such reconstructions were used to discuss the structural setting of East Antarctica with respect to the setting of Southern Australia and showed that the magnetic data indicate the continuation of the Australian geology under the ice of Antarctica^10–12^. That link between Antarctica and Australia has been studied in detail using aeromagnetic data of comparable quality. Studies on the link between Antarctica and Southern Africa, however, have often had a focus on Dronning Maud Land on the Antarctic side or relied on low-quality global magnetic compilations outside Antarctica^13–15^. Similar observations can be made for the link between India and Antarctica, which has been studied albeit without using magnetic data^16^. A reason for this is that aeromagnetic data for the Indian subcontinent are not openly available and in state-of-the-art global compilations models like EMAG2v3^17^ are indicated to be of low quality.

Resolution and accuracy are in general an issue when using global magnetic anomaly compilations to derive tectonic interpretations and supercontinent linkages. For example, EMAG2v3 has a nominal resolution of 2 arc-minutes, but data content is highly heterogeneous over the globe. That relates to accuracy and resolution, but also the treatment of the long-wavelength content that is notoriously unreliable. The maximum wavelength contained in any dataset is limited by the survey extent and often one refers to a spectral gap between satellite and aeromagnetic data. To address this spectral gap, in Australia a number of long-haul flight lines had been acquired^18–20^. Still, the long-wavelength range in the most recent release has been replaced by satellite data due to their increased accuracy. A number of studies have made use of recent releases of the satellite lithospheric magnetic field to replace the long-wavelength part of aeromagnetic surveys with satellite data^21,22^. For example, in the Circum-Arctic region, the long-wavelength aeromagnetic data were replaced with satellite data using a cut-off filter of 400 and 330 km^22^. This cut-off relates to the maximum spatial resolution of the early generation of satellite models^23–25^ and corresponds to the maximum reliable wavelength of typical aeromagnetic surveys.

The resolution of satellite-derived magnetic field models is limited by the orbit height, instrumentation and mission period. Since 2013, the Swarm satellite mission from the European Space Agency has been continuously measuring the Earth’s magnetic field. The mission consists of three-satellites at different altitudes and with changing orbit inclinations^25,26^. The addition of these data to the data from previous satellite missions (e.g. CHAMP, Oersted) helps to increase spectral accuracy and appears in the Swarm-derived lithospheric field model LCS-1 providing reliable wavelengths in some regions to 250 km^25^. However, in Polar Regions, the noise in satellite data is greater than at mid-latitudes^27^. Due to the increased noise, the lithospheric field estimates are less accurate for wavelengths larger than 300 km wavelength.

Here, we conformed the available aeromagnetic compilations for Antarctic and its neighbouring continents Australia and Southern Africa to satellite data. This process results in a homogeneous data representation across the heart of Gondwana. We discuss the value of the conformed data and the satellite data itself by linking up anomalies between Southern Africa, Australia and East Antarctica. In addition, we look at the satellite data link to India to its Antarctic counterpart.

## Conforming satellite magnetic and aeromagnetic data

**Figure 2:**
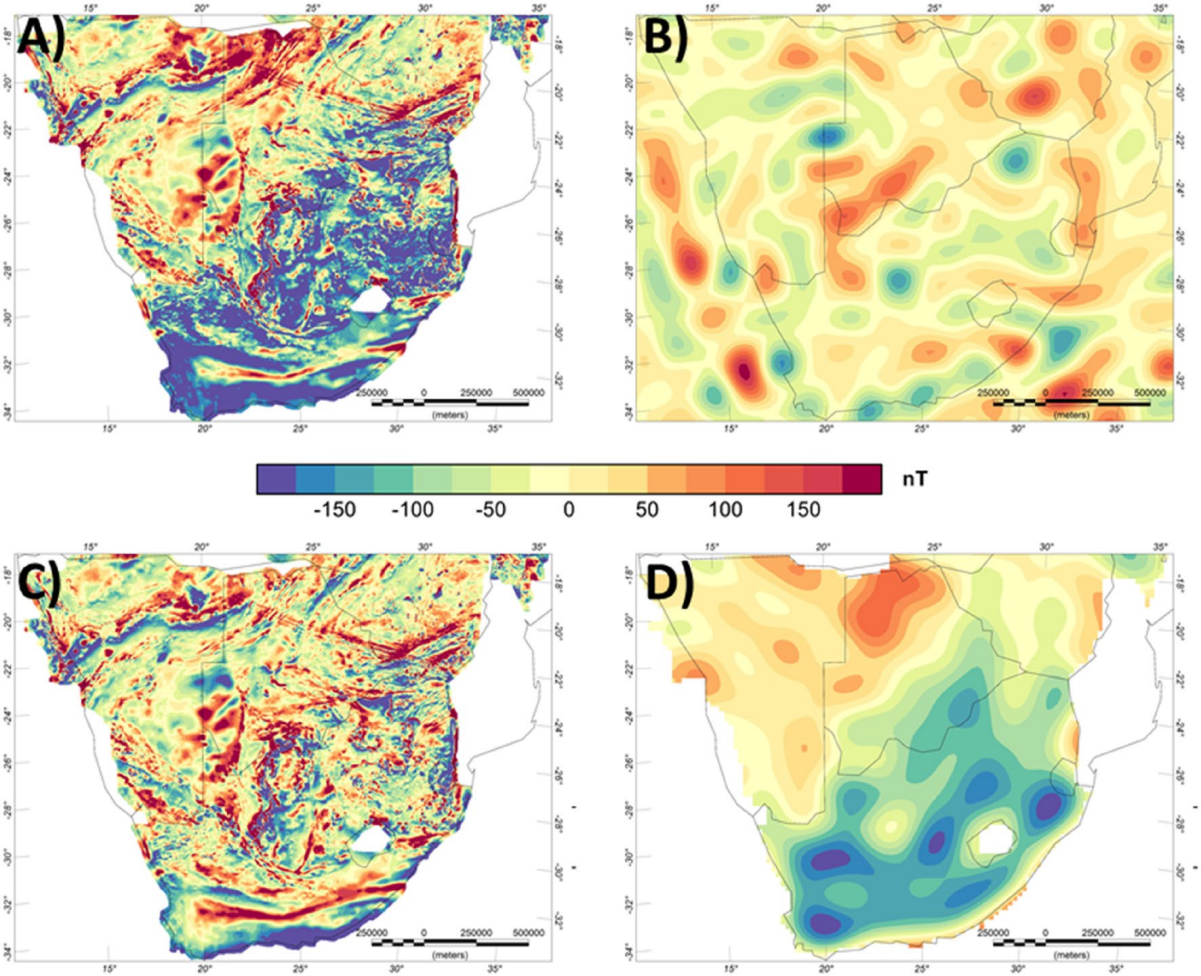
Lithospheric magnetic anomaly of Southern Africa. (**A**) Original aeromagnetic compilation^29^, (**B**) Satellite-derived model LCS-1, (**C**) Conformed aeromagnetic and satellite model. (**D**) Difference of (**A**,**C**).

To illustrate the different steps in homogenising the aeromagnetic data, we choose the area of Southern Africa (Fig. 2). Two aeromagnetic grids cover the area of South Africa, one made publicly available in 2000s^29^ and the second, an updated version, released in 2019^30^. In the original compilation a difference in the main level of > -100 nT can be observed between South Africa and its northern African neighbours (Fig. 2A), while in the latest release South Africa is shifted by ~+50 nT (not shown here).

The difference in the level could be misinterpreted as reflecting fundamental variability in crustal-scale geology or thermal state. E.g. the long to mid wavelength field is often used to estimate the Curie isotherm^31^, but can also relate to differences in crustal architecture^32^, upper mantle magnetisation^33^ or a combination of thermal and structural properties^34^. For this area, the pattern partially correlates with some of the main tectonic features of the area such as the boundaries of the Kaapvaal Craton to its surrounding. An indication, that this feature here is instead an artefact of processing comes from the fact that the shift values for the two generations of compilation have an opposite sign.

Therefore, we replace the long-wavelength part of the original 2000s Southern Africa aeromagnetic compilations with the satellite model LCS-1 (Fig. 2B). For this, we use an equivalent layer approach and replace the data up to spherical harmonic degree 130 (corresponding to ~300 km wavelength) with satellite data (see Data and Methods for more details). The resulting conformed grid is spectrally consistent and still contains the high-frequency information of the original compilation.

The long-wavelength part of the aeromagnetic compilation, if accurately estimated, is expected to be similar to satellite derived data. However, the long-wavelength part shows differences of more than -100 nT for South Africa and considerably less differences for the countries to its north as can be seen in the correction applied to the data (Fig. 2D).

As mentioned, for South Africa, an updated aeromagnetic grid is as well available^30^, having an apparent higher resolution, due to the inclusion of more recent surveys, and with an opposite sign shift of 50 nT for the long-wavelength in South Africa. Conforming this grid to the satellite model results again in a long-wavelength trend correction and in a homogenous anomaly map that still reflects the major crustal domains of Southern Africa.

We applied the same procedure to the Australian and Antarctic datasets (see Data and Methods). For Australia, the differences in the long-wavelength range are relatively small. The differences result from the use of a different satellite model as compared to original Australian compilation, where the long-wavelength part was replaced by the satellite model MF-7^24^. Here, we replace instead the long-wavelength components with the more recent LCS-1 model for improved consistency between the compilations for the individual continents we examined.

**Figure 3:**
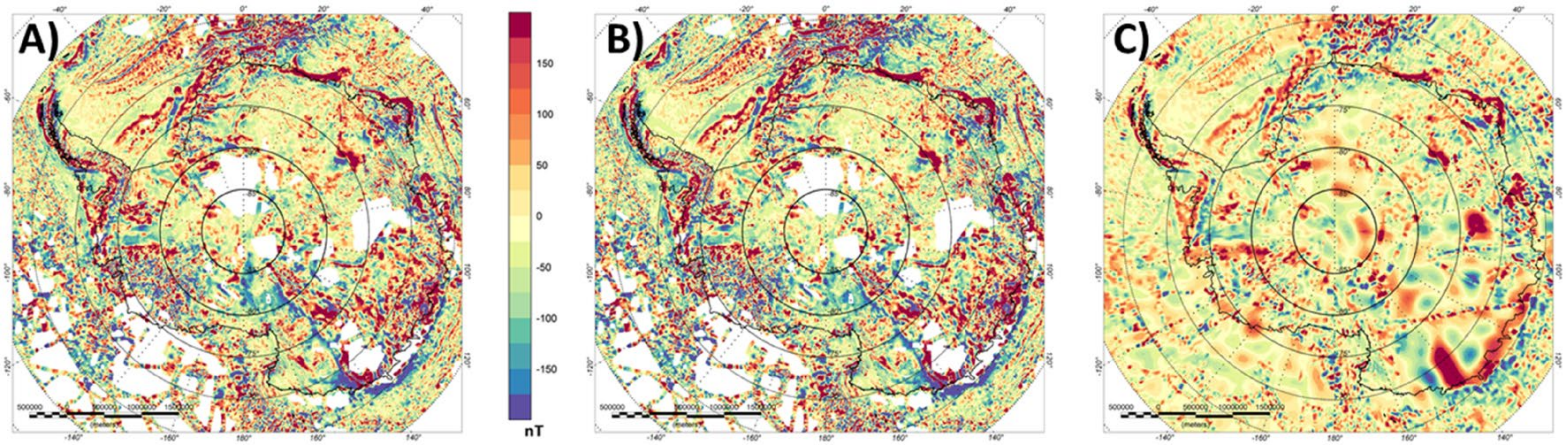
Lithospheric magnetic anomaly for Antarctica. (**A**) Original ADMAP-2 compilation^35^. (**B**) After conforming ADMAP-2 to the satellite model LCS-1. (**C**) EMAG2v3^17^.

More interesting are the differences for Antarctica. For the ADMAP-2 compilation all surveys have been corrected for the same geomagnetic reference model, but no correction for satellite data has been applied^35^. Nevertheless, the long-wavelength component of ADMAP-2 still corresponds very well with the satellite-derived model. Conforming the Antarctic aeromagnetic compilation to the satellite model leads to sharpening of some anomalies with the main differences between satellite and aeromagnetic data in the long-wavelength range being correlated to the line density and general data coverage (Fig. 3). In areas where the aeromagnetic data coverage is denser, the location of the anomalies are similar, but shapes and extents may vary.

Unfortunately, not all of the continents surrounding Antarctica, have aeromagnetic compilations in the public domain, e.g. India. Therefore, one might consider using data from global compilations to fill the gaps. In Fig. 3, we compare the conformed satellite-aeromagnetic anomaly map to the global model EMAG2v3 for Antarctica. The comparison shows that most of the details seen in our satellite-corrected aeromagnetic data compilation are less clear in the global compilation. EMAG2v3 comes with a data quality measurement that shows the dataset does not contain consistent high-quality measurements for this area. It is evident that EMAG2v3 is only a smoothed representation of the aeromagnetic datasets and some of the linear features appear blurred and do not show the strike directions.

For India, the quality of the data in EMAG2v3 is even worse^17^ and hence any correlation of the features to East Antarctica is even more complicated. Another issue is the direction of the inducing main field. Induced magnetisation is commonly assumed to be the dominant magnetisation over the continents, while remanent magnetisation dominates over the oceanic plates^36^. The direction and strength of the magnetic main field changes from the poles to the equator. While the strength of the field governs the amplitudes of the anomalies, the inclination of the field governs the shape and location of the anomalies. For a vertical field, the peak anomalies are located over the source body and side slopes are small. For an inclined field, a typical magnetic dipole anomaly is observed, where none of the anomalies are necessarily located directly over the source. For Polar Regions, such as Antarctica, this directional effect is often ignored, but for inclinations less than 70 degrees, the changes become more important.

While the inclination does not represent the inclination during break-up, it implies that simple reconstructions show rock formations adjacent to each other that actually have a completely different orientation in the Earth magnetic field because their magnetic field is based on the present day continent layout. The inclination in Southern Africa and Australia is around 50 degrees and increases to 90 degrees in Antarctica. India is located slightly north of the magnetic equator (where inclination is zero). Therefore, after conforming the compilations to a common satellite model, we performed a reduction to the pole considering the variable direction of the magnetic field (Fig. 5 and see Methods section). However, this correction should consider the orientation of the magnetic field during the time of acquisition. Such information is next to impossible to retrieve for all the surveys acquired over more than 60 years. We applied the correction only to the satellite model for which a reduction to the pole (RTP) with a common datum is straight forward. Common spectral methods for reduction to the pole fail for satellite data. Instead, we calculated a RTP satellite model with an equivalent source method. This method also does not suffer from the numerical instability at the magnetic equator as Fourier-based methods do, but as it is computationally expensive was not used for the aeromagnetic data sets.

## Discussion

**Figure 4:**
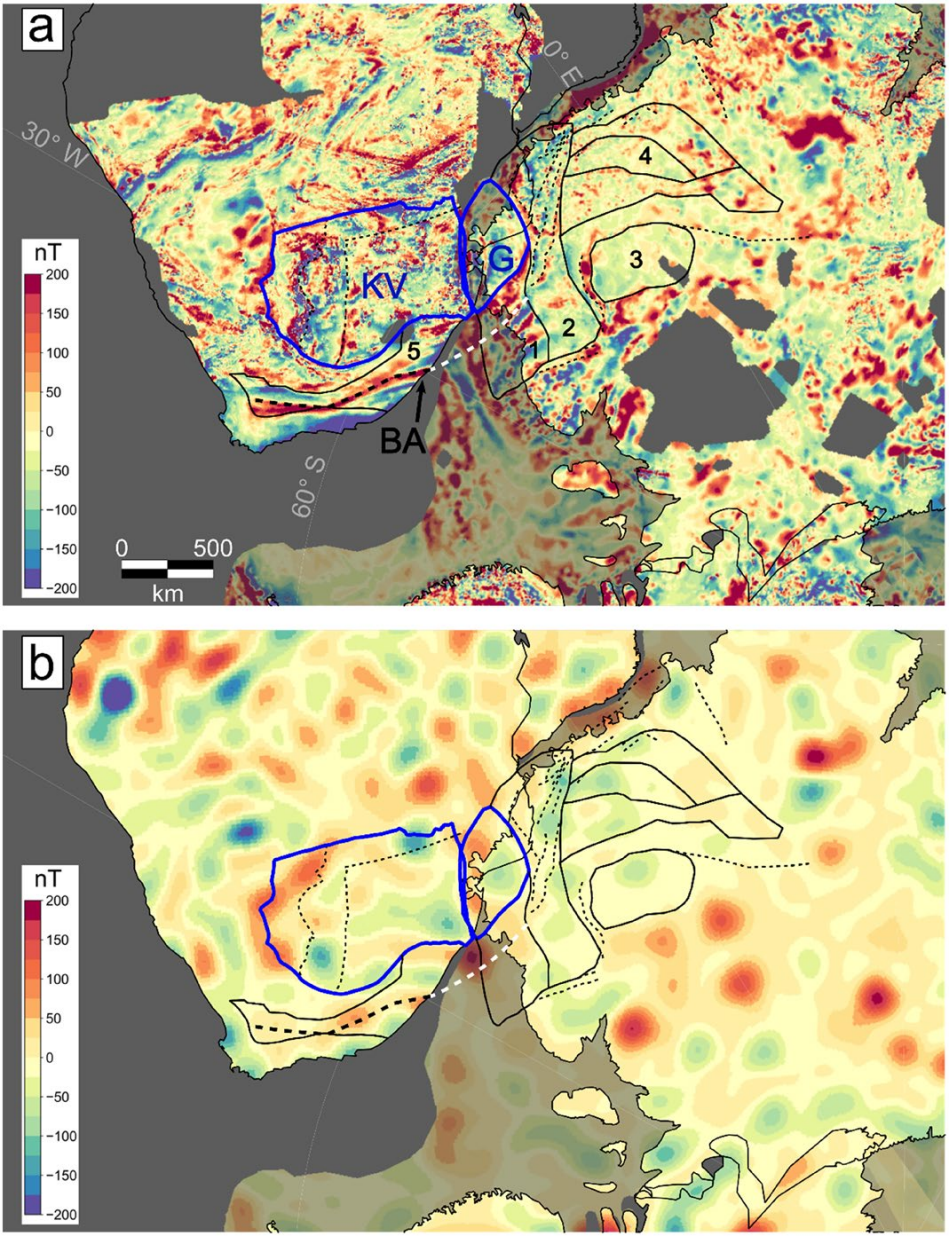
Zoom in on the South Africa-Antarctica connection. (**a**) Combined aeromagnetic-satellite model. The connecting cratons are marked as blue contours. KV: Kaapvaal, G: Grunehogna Craton. Beattie Magnetic Anomaly (BA) is indicated as thick dashed line. Solid black contours represent selected tectonic units of Antarctica older than 200 Ma. 1: Natal type terrane boundaries. 2: Maud Belt, 3: Valkyrie Craton, 4: Tonian Antarctic Super Terrane (TOAST). Contour 5: Natal terrane. Dashed lines indicate several fracture zones in South Africa and Dronning Maud Land, White dashed line: Continuation of the Beattie Magnetic Anomaly, (**b**) Satellite data LCS-1. Both data sets have been reduced to the pole and are represented at 5 km height. All data sets are rotated back to 200 Ma. Semi-transparent anomalies indicate aeromagnetic anomalies on the continental margin.

**Figure 5:**
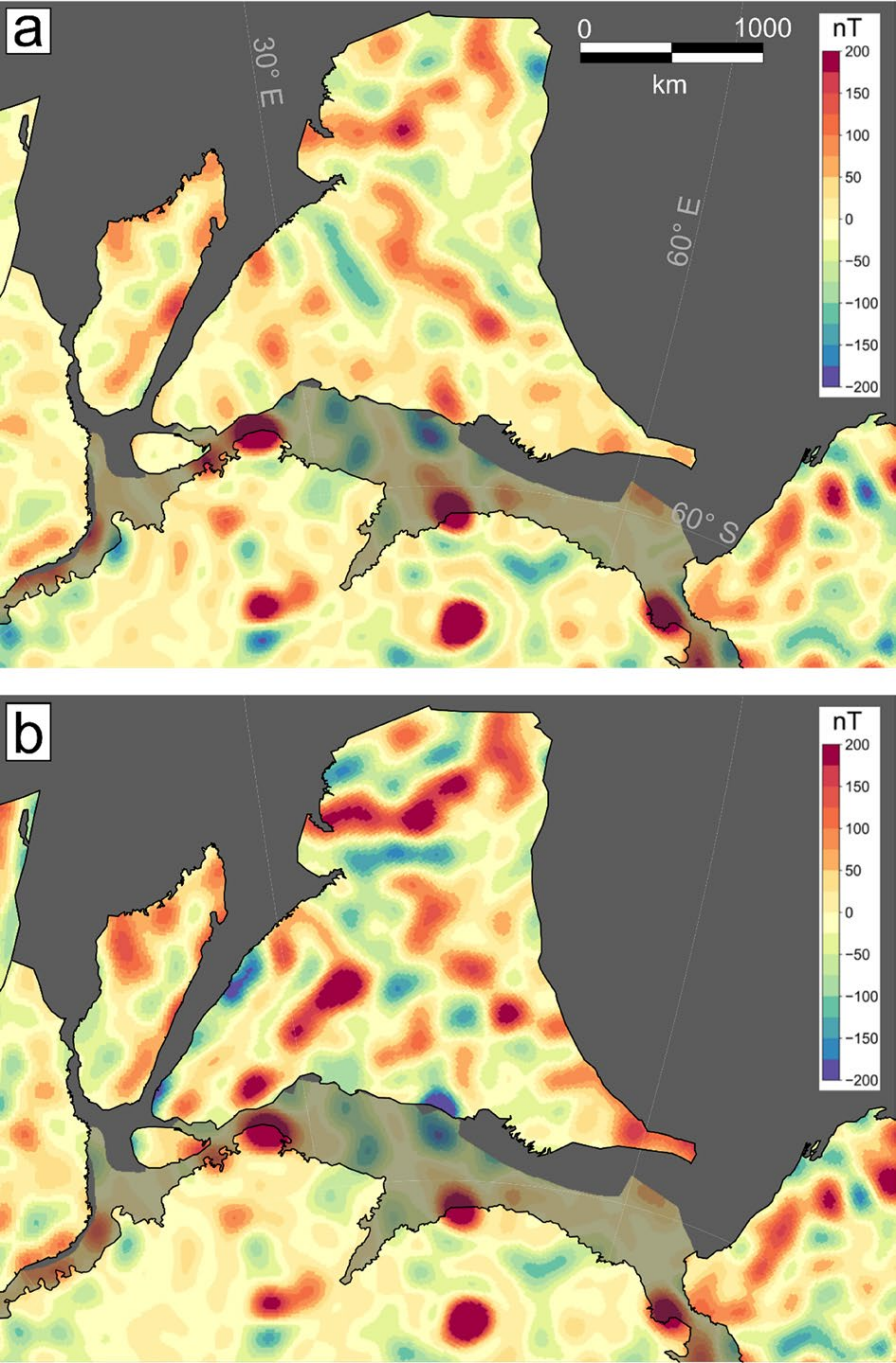
Zoom in on the India-Antarctica connection. (**a**) Original satellite mode LCS-1, (**b**) RTP corrected satellite model. Both data sets are represented at 5 km height. All data sets are rotated back to 200 Ma. Semi-transparent anomalies indicate aeromagnetic anomalies on the continental margin.

Two things are of interest in interpreting the conformed aeromagnetic and satellite data: (1) the internal structure of the continents, especially East Antarctica, and (2) the link to its adjacent parts in a Gondwana framework. Figures 1, 4 and 5 show the results in a pre-Gondwana break-up configuration ca 200 Ma ago based on a deforming plate model^5^.

The area, where the integration of satellite and aeromagnetic data has changed the most is Southern Africa. Figure 4 shows a zoom to the link between South Africa and East Antarctica. It has been already discussed that the Beattie anomaly can be linked to an anomaly with similar amplitude and trend in Dronning Maud Land, Antarctica^37^ that sharply terminates along the western orogenic front of the East African Orogen^38–40^. Recently, it was suggested by geological-petrological modelling that the Beattie anomaly is linked to a shear zone near the southern boundary of the Natal terrane^41^. The anomaly is part of a family of anomalies, one of which correlates with outcropping shear zones in the Mesoproterozoic Natal belt for which a complex remanent magnetisation was suggested^43^. However, the corresponding Antarctic anomalies have a higher amplitude (Fig. 4), due to the fact that the corresponding sources on the South African side are covered by sediments of the Karoo basin. In the satellite data, a weak imprint of the Beattie anomaly is visible, which confirms its regional significance (Fig. 4).

The satellite data show their potential in illuminating the link between India and East Antarctica (Fig. 5). In the original satellite data, the Indian-Antarctic link is not very pronounced, but after the RTP correction, the anomalies line up, indicating a continuation of the Indian geological provinces under the Antarctic ice. Satellite data are limited in their ability to resolve local structures, but are expected to reflect the major tectonic setting. The match-up of the India and Antarctic pieces suggest that the sources are upper crustal Precambrian basement that has not been significantly altered by continental break-up.

However, future more detailed analysis should consider the importance of remanent magnetisation, as many of these rock formations are billions of years old and gained parts of their magnetic orientation during emplacement or formation when the Earth's magnetic field had a different orientation. Ideally, to increase the correlation of the Indian continent to Antarctica, one would need to process the aeromagnetic data for India in the same manner as for the other continents, if the survey data became available.

## Conclusions

Aeromagnetic data are important when discussing the tectonic setting of the continents, especially in a plate reconstruction. However, aeromagnetic compilations should be conformed to satellite data to be spectrally consistent, especially when these compilations are based on multiple surveys acquired over a long time period. The increased resolution and accuracy of satellite data allows confident identification of the main tectonic signatures in the data sets. In this study, the higher resolution of the conformed compilations further aids the interpretation of the links between East Antarctica and its former Gondwana neighbours. That is clearly seen both in the well-established link between Australia and Antarctica and the less-explored link between the interior of Southern Africa and Antarctica. RTP satellite data show furthermore the huge potential for reinterpreting the link between India and East Antarctica, when aeromagnetic data over the Indian subcontinent become available.

Less important here is the exact geometry of the margins and break-up, but that properties from the interior of the continents can be linked to their previous neighbours. Here, we emphasis this for East Antarctica. A natural next step would be to apply multivariate analysis^3^ adding the magnetic data. That would allow definition of lithospheric domains for East Antarctica in line with the tectonic setting of Australia, Southern Africa and possibly India. This furthermore could help to interpolate other sparse measurements into East Antarctica or to define statistical correlations which can be used for predictions. A current application is deriving geothermal parameters under the Antarctic ice^45^. Guided interpolation of geothermal heat-flow values using the magnetic field is possible, as well as cluster analysis and machine learning approaches, where statistically correlation are exploited for improved predictions.

## Data and methods

**Figure 6:**
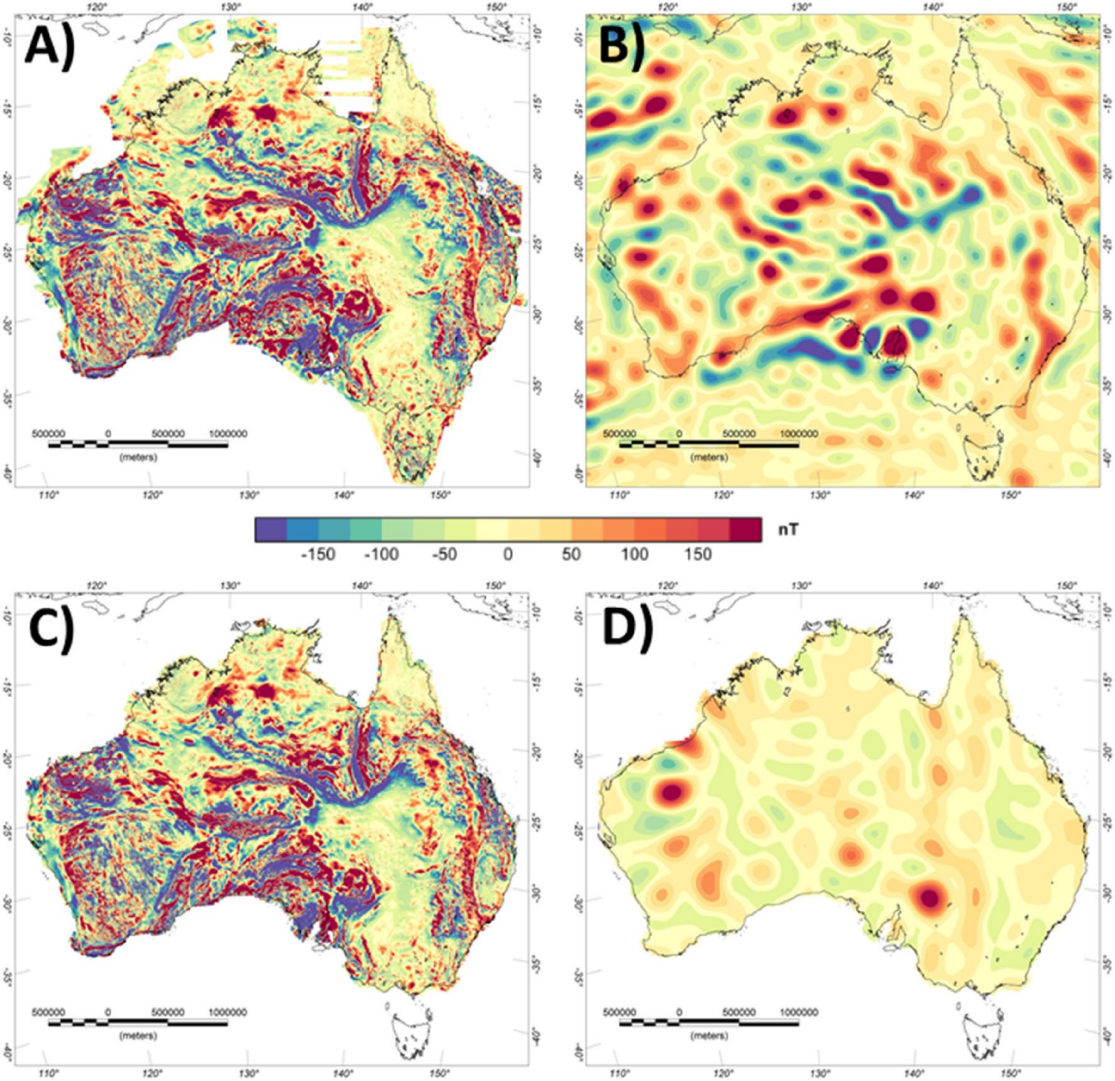
Lithospheric magnetic anomaly of Australia. (**A**) Original aeromagnetic compilation^20^, (**B**) Satellite-derived model LCS-1, (**C**) Conformed aeromagnetic and satellite model, (**D**) Difference between (**A**,**C**).

**Figure 7:**
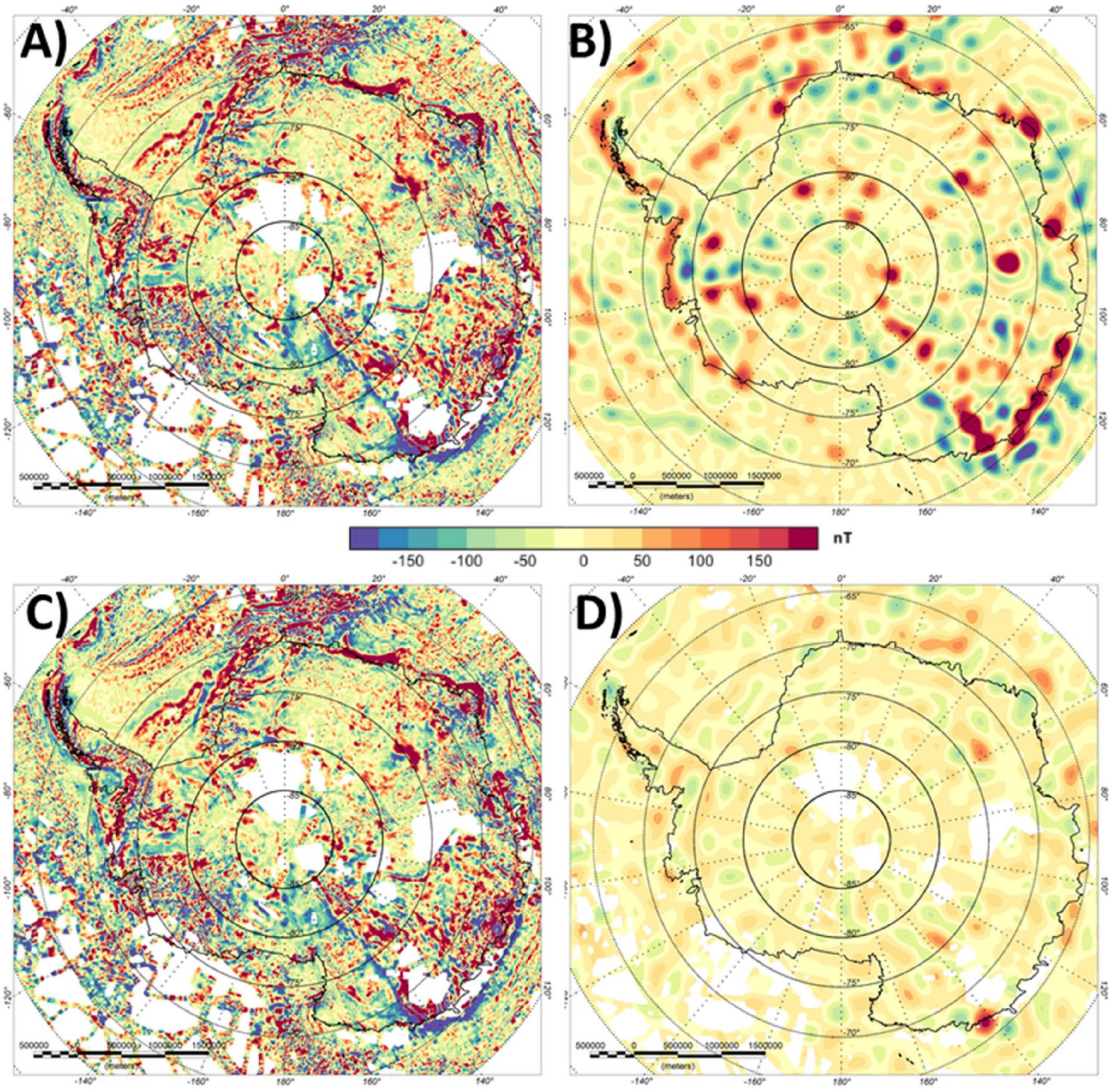
Lithospheric magnetic anomaly of Antarctica. (**A**) Original aeromagnetic compilation^35^, (**B**) Satellite-derived model LCS-1, (**C**) Conformed aeromagnetic and satellite model, (**D**) Difference between (**A**,**C**).

Australia, Southern Africa and Antarctica are three of the areas of the world with particularly comprehensive aeromagnetic data coverage (see Figures 2, 6 and 7). In Australia and Southern Africa, widespread high-resolution aeromagnetic data acquisition has primarily been motivated by mineral resource mapping^19,29^, while in Antarctica the focus has mostly been on systematically exploring subglacial geology and defining large-scale crustal architecture^13,35,44^. Therefore, the survey parameters (e.g. survey height and line spacing) typically adopted in the three continents are quite different.

Over Australia more than 800 individual surveys are nowadays included in Geoscience Australia's National Airborne Geophysical Database (NAGD) that contains more than 31 million line kilometres of total field magnetic intensity data. Since 1990, surveys have usually been conducted with flight line spacing of 400 metres or less^19^. To reduce long-wavelengths errors, high-altitude airborne traverse data were used, which significantly improved the intermediate wavelengths from 100 to 500 km. For edition 5 of the magnetic anomaly map for Australia, which is used here, the long-wavelength part (>1000km) has been replaced with the satellite magnetic model MF-6.

Systematic data acquisition in Antarctica started as early as the International Geophysical Year 1957–1958. A wealth of modern airborne geophysical surveys, including airborne gravimetry and aeromagnetic data acquisition over previously largely unexplored Antarctic frontiers, such as the Gamburtsev Subglacial Mountains^46^ and Wilkes Land in East Antarctica^11^ was stimulated by the International Polar Year 2007/2008. The first Antarctic magnetic anomaly compilation (ADMAP‐1) was produced in 2001 from more than 1.5 million line kilometres of shipborne and airborne measurements^47^. This was succeeded in 2018 by the second-generation Antarctic magnetic anomaly compilation (ADMAP-2) that includes more than 3.5 million line‐km of aeromagnetic and marine magnetic data that more than doubles the initial near‐surface database^35^. Large-scale international aeromagnetic exploration in Antarctica has continued since ADMAP-2, with major more recent surveys flown e.g. over the Recovery and South Pole frontiers^48,49^.

For Southern Africa (Fig. 2), two modern compilations exist from the Council for Geoscience, Republic of South Africa, who began collecting and compiling aeromagnetic data in the 1970s. Similar programs of collecting aeromagnetic data throughout the Southern Africa region have resulted in nearly complete aeromagnetic coverage of South Africa, Namibia, Botswana and Zimbabwe. The Council for Geoscience has compiled all of these magnetic data available in southern Africa and has gridded the data at one km grid spacing ^29^. Because the data have a relatively large line spacing (1 km), they were initially only used for large scale mapping, mainly for mapping the Kaapvaal Craton boundary, lithology identification and for defining important linear magnetic features^50^. An update for the Republic of South Africa is now available^30^. The long-wavelength part between the two compilations is quite different, while the short-wavelength part is very similar, again confirming the use of a common reference model. Quantitative interpretations of the higher resolution data concentrated on individual targets such as the Beattie magnetic anomaly^41,51,52^.

The satellite model for the lithospheric magnetic field is LCS-1^25^. LCS-1 contains the spherical harmonics from degree and order 15-185. The lower order spherical harmonics are associated with the main (core) field and omitted from the lithospheric field part. In comparison to previous models, LCS-1 represents the field to a high spherical harmonic degree by providing coefficients to 185 corresponding to ~220 km wavelength. Based on the data from pre-Swarm satellite mission data only, earlier models limited the representation to degree 70 (~600 km), 85 (550 km) or 130 (300 km). For LCS-1, the spectral range to degree 130 is globally consistently estimated, while for the higher spherical harmonics, especially in Polar Regions, the noise increases, making the representation less accurate. Still, for Australia there is lithospheric signal contained in the higher spherical harmonics^25^.

## Conforming aeromagnetic compilation to satellite model

To homogenize the data, we applied an equivalent dipole layer approach coupled with spherical harmonic representation^54^. The reason for using an equivalent source method is, that the Fourier based methods result in artefacts when applied to global long-wavelength satellite data and suffer from instabilities at the magnetic equator. Equivalent sources do not suffer from this instability, but require extensive computational resources. Hence for the aeromagnetic surveys a simplification has to be made.

1. For estimating the magnetic parameters of the equivalent dipole layer, we used BiCGSTAB iterative inversion method. For the satellite data, we located the equivalent dipole layer at 30 km beneath the reference level with a horizontal spacing of 25 km corresponding to the data spacing. These values were found to best represent both the spectral content at an observation height of 400 km and near surface. The field was inverted using the direction of the main field model CHAOS-6^53^ at each dipole location. No amplitude correction was applied. CHAOS-6 was also used as reference model for LCS-1.
2. From the equivalent dipole layer, we forward calculate the RTP model at 5 km ellipsoidal height for a vertical field and a constant field strength.
3. For the aeromagnetic data, we also applied an equivalent dipole layer representation, but for computational reasons, first performed a simple resampling of the data data to ~22 km (0.2 degree equiangular) resolution for Australia and Southern Africa and ~44 km (equidistant) for Antarctica. This step did not affect the long-wavelength content of the data, but obviously limited the short-wavelength content. The equivalent sources were again located at 30 km beneath the reference level and were calculated using CHAOS-6 as main field.
4. Next, we estimated the spherical harmonic coefficients from the equivalent layer for both the satellite and aeromagnetic data by calculating the coefficient for each dipole and summing up their individual effects. Spherical harmonic coefficients have been calculated to degree and order 180 for satellite data and 720 for the aeromagnetic data sets
5. For spherical harmonic coefficients up to degree and order 130, the difference between the satellite and aeromagnetic data has been calculated and used to correct the original aeromagnetic data.

## Plate-tectonic illustrations

Figures 1, 4,5 and the movie in the supplementary material have been made with GPlates (http://www.gplates.org/) using its global reconstruction files. The supplementary animation provides an example for the last 200 My of plate tectonics centred over Antarctica. The movie illustrates with the conformed aeromagnetic data and satellite data as infill, where no aeromagnetic data are available, the link between Antarctica and the adjacent continents. The plate-tectonics illustration was done in GPlates (https://www.gplates.org/).

## Supplementary Information

Supplementary Information 1.

Supplementary Video 1.

## Data Availability

The aeromagnetic data are available at: South Africa: http://www.geoscience.org.za/index.php/2019-03-13-12-40-41/publications/284-geophysical-data. Australia: http://www.ga.gov.au/news-events/news/latest-news/latest-editions-of-magnetic-anomaly-grid-and-radiometric-map-released. Antarctica: https://doi.pangaea.de/10.1594/PANGAEA.892724. The satellite model LCS-1 can be accessed at: http://www.spacecenter.dk/files/magnetic-models/LCS-1/. The reprocesses grids with 0.1 degree resolution can be accessed here: https://www.3dearth.uni-kiel.de/en/public-data-products. Full resolution data sets can be provided on request by the authors.
