# Supplementary material for: East Antarctica magnetically linked to its ancient neighbours in Gondwana
Source: Sci Rep. 2021 Mar 9;11:5513. doi: 10.1038/s41598-021-84834-1 (PMC7970907; doi:10.1038/s41598-021-84834-1)
Supplement: Supplementary file 1 — Supplementary Information 1. [file 41598_2021_84834_MOESM1_ESM.docx]

Supplementary Material for “East Antarctica magnetically linked to its ancient neighbours in Gondwana “

Jörg Ebbing1*, Yixiati Dilixiati 1, Peter Haas1, Fausto Ferraccioli2,3, Stephanie Scheiber-Enslin4

*Correspondence to: Joerg.Ebbing@ifg.uni-kiel.de

1 Institute for Geosciences, Kiel University, Kiel, Germany

2 OGS, Trieste, Italy

3 British Antarctic Survey, Cambridge, UK

4 Witwatersrand University, Johannesburg, South Africa

Supplementary File 1: “Aeromag Gondwana SupplementaryAnimation.mp4”
